# Allegheny Medical Society

**Published:** 1891-09

**Authors:** 


### ALLEGHENY COUNTY MEDICAL SOCIETY.

### SCIENTIFIC MEETING, JUNE 16, 1891.

T. D. DAVIS, M. D., President, in the chair.

DR. BLUME: Vomiting of Pregnancy. (See page 400.)

DR. LEMOYNE: I agree with the doctor that in a great many cases vomiting is largely due to abnormal conditions of the parts. I had the same experience with obstinate vomiting in this condition, and found it often was relieved by the correction of some erosion or displacement, although I do not believe that that always caused the condition. The doctor has very wisely laid a great deal of stress upon the matter of careful examination of the parts where these conditions exist, with the view of ascertaining and removing any such abnormal conditions.

DR. LANGE: An excellent paper the doctor has given us. It deserves our thanks. It is very comprehensive and thorough, and there is nothing in it to which any one can raise any objection, except perhaps this—the rectification of displacements of the uterus during pregnancy by the use of pessaries. That, in my experience, is impossible. In an impregnated uterus, and perhaps in a uterus which is not impregnated, I think that it is beginning to be thoroughly understood that the pessary is a means of absolutely no value, sometimes of great discomfort, and occasionally of some danger. The only pessary which may perhaps be sometimes of service is the ordinary balloon pessary, a rubber globe which is ballooned up by inflation with air or water, This, in my experience, prevents the descent of the uterus in some cases. The stem pessary of Dr. Kinloch may also be an exception to the rule that pessaries are useless for uterine displacement. This can be used, of course, only in the unimpregnated uterus, and may deserve consideration in flexions. Among the remedies which the doctor has mentioned are the most valuable, but I fail to hear calomel. Calomel has been, in my experience, a most valuable remedy. In proof of this, I had a lady under my care who had twice suffered abortion at four months, at the hands of most eminent gentlemen in the profession, because of pernicious vomiting, and their conclusion was that she would die. At her third pregnancy, she and her husband again concluded that she would die, and she certainly looked like it. Her adipose tissue had disappeared, her muscular system had atrophied, her belly was distended and tender, she was blanched, her pulse was small and rapid, she had an elevated temperature, and her tongue was red and dry. I gave her calomel—one-eighth grain every three hours. In forty-eight hours she was able to retain some food; in two weeks she left her bed, still sick, still vomiting, but able, despite her vomiting, to take a sufficient amount of food to maintain life and improve her condition. That is one instance where calomel exerted a very marked good effect and this was not due to any organic disease of the stomach. Sufficient proof of this is the fact that before her pregnancy she was always well, and the same was true before her previous pregnancies. She is the wife of a druggist in this city. I give calomel frequently in the vomiting of pregnancy, but in the case related it strikingly exhibited its power. The doctor mentioned a remedy with which I have no experience in the vomiting of pregnancy, but of which I have heard much good. I have always understood that the introduction of that method belonged to a Pittsburger, namely, Dr. M. O. Jones, of Wylie avenue, who informed Dr. Marion Sims of it; the latter teaching it in Paris in 1870. I have always understood that dilatation of the cervix and the separation of the membranes a little way up, which is said to be a very excellent remedy for the ordinary sickness of pregnancy, is the method of Dr. Jones.

DR. DAGGETT: The doctor speaks of increased temperature being one of the symptoms of pernicious vomiting. Is that one of the signs which arise before death?

DR. BLUME: In my case, after about two weeks had passed fever commenced, and after another week had passed, the woman still in bed, her fever went up to 103 and remained at that height for several weeks, and this case recovered without any medicine whatever, having nothing but absolute rest. She was in bed about six weeks.

DR. BATTEN: I have had too cases of severe vomiting of pregnancy. One was in the person of an unmarried woman At first I could not imagine (she was supposed to be a virgin) what the trouble could be. She was emaciated and her eyes were sunken; after two or three days my suspicion was aroused. There was no fever. The case went on for two weeks under my treatment with all the remedies that I knew of, excepting local applications, and from the fact that the young woman made the request that I keep her mother in darkness as to the cause of the trouble, I was prevented from making any local applications. But after exhausting everything I returned to blackberry brandy and stopped all medicine; after giving her the second dose the vomiting ceased and she recovered after taking the remedy some three weeks. Another case I had in 1882. A woman had seven children and never had any vomiting whatever; in this case the vomiting appeared at the end of the first month and continued right along to the end of the fifth month, and all remedies seemed to have no control whatever of the trouble. However, at the end of the fifth month, after I had given up the treatment, she was not so prostrated as the other case. It was a twin pregnancy, and when that woman was delivered one child was fully developed and another was dead, having died evidently at about the fifth month, about the time the sickness left her. The dead fœtus was in the neighborhood of five or six inches long. The woman ran on to full time.

DR. CONNELL: One point the doctor alluded to, the use of nitrate of silver. My attention was first called to the use of this remedy by Dr. Jones, whose name has been mentioned, and I think to him belongs the credit of the introduction of nitrate of silver treatment. In the *Journal of the American Medical Association*, of two years ago, there was a paper written by a gynecologist or obstretrician of Washington, whose name I cannot now recall, and in reply to that, Dr. Jones gave his experience in the use of nitrate of silver, and alluded to having spoken to Dr. Sims about it. Dr. Jones used that many years ago, and it is only a few years since the attention of the profession was called to it. I have used it and had better results with the application of the nitrate of silver than with any other remedy.

DR. HUSELTON : I want to say a word in defence of the pessary. I think we have displacement of the uterus in the early stages of pregnancy. I also believe it is possible to rectify them and maintain them in proper position by the use of Thomas' retroversion pessary. I have certainly replaced retroverted uteri and maintained them in position with this pessary. With regard to treatment, as has been properly stated, I do not know of any one remedy that will relieve all cases. I remember one very aggravated case I had a number of years ago, in which I tried everything that I could think of, which I afterwards succeeded in relieving entirely with a large dose of chloride of potassium.

DR. GREEN : I have no criticism to offer on the paper. I wish to relate a case of vomiting during pregnancy which came under my observation on the sixth of this month. The lady was three month's pregnant. She had been treated by two or three physicians previous to her application to me. I do not know what remedies she had taken, but no relief had been afforded by any of them. I found her very much prostrated, and for five or six weeks she had had difficulty in retaining food sufficient to afford nourishment; she had not rejected all the food and was still able to go about. An examination of the uterus discovered it to be displaced, retroflexed and apparently bound down with adhesions, and abortion was about to take place. There was evident hemorrhage, which had begun some time the previous night. She miscarried on the second day after I saw her. Again, I wish to say a word in regard to the use of the pessary. Whilst I have no doubt that the pessary may in certain cases do some damage, I have seen what I supposed was benefit, what I supposed was very great benefit, in the use of the soft pessary ; especially in cases coming under the class where displacement can be assigned as the cause, I have seen permanent relief. I wish to say a word about the damage sometimes done by pessaries. About six weeks ago I delivered a lady at term. On making examination, I found a common ring pessary, hard rubber. I said: "How long have you worn this?" She replied, "About five years." Six years previously she had given birth to a child and immediately after that this pessary had been placed in the vagina and remained there ever since. I removed the pessary, and saw that it had done no harm. This, I presume, does not very often occur. It shows that the pessary does not always do very great damage.

DR. WERDER: I have no personal experience in this matter. I have had a large number of pregnant women under my observation, but have never seen a case of serious vomiting of pregnancy. There is no doubt that vomiting of pregnancy is a reflex neurosis—of course that is not saying very much—but I think in a large number of cases it is a form of hysteria. A number of cases have been reported which were treated as hysteria and got well under that treatment; before that, other methods of treatment were employed without any benefit whatever. I think there is no doubt that a large number of cases are hysterical in their character. In regard to pessaries, I have no doubt that pessaries are used very much, and very many women would be far better without them, but there are exceptions to that. I think a good many women would not feel comfortable without the pessary. If the uterus is properly replaced and in normal position and a pessary is introduced, where there is no inflammatory condition of the pelvis, in many cases it does a great deal of good. Many of these women are very comfortable wearing a pessary for months, and women come to me with pessaries where there is no flexion at all, where there is no displacement of the womb, and sometimes there is a displacement, but it has not been reduced at all, the uterus is retroflexed just the same as it was before the pessary was introduced. In pregnancy I have had probably two or three cases of this class, and am certain that these women suffered before the uterus was put in position and before the pessary was introduced, and the benefit they derived from the pessary was also very great.

DR. DAVIS: Dr. Blume certainly covers the ground in every particular very thoroughly, and there is one point that he dwelt on and that is, as he said very aptly, you would think that the diagnosis of pregnancy was a very easy thing, and yet I believe this is an important point of the question for discussion to-night. I believe that on that hangs the treatment, and that is the reason why such various treatments are recommended, and why in some cases one treatment acts very favorably and another treatment is of no benefit. I think it stands to reason that if pregnancy is the cause of this vomiting, calomel would have very little effect. I think it stands to reason that if there is no (or very little) trouble of the uterus, but if there is a disease of the stomach, calomel would be very beneficial. And therefore, while in one case I would expect benefit from it, in the other, it would be *nil*. On one occasion I was called to see two ladies, neighbors, both suffering with morning sickness of no aggravated form, but enough to give them great discomfort. To the one I prescribed to the best of my judgment what was wanted, bromide of potash. In the other I prescribed hypophosphites in an acid solution. I remember distinctly they were given in quite large bottles; they each used about half of the bottles, which were given within a day or two of the same time, and were none the better, but rather grew worse. Talking, as neighbors will do, over the back fence, they compared notes and traded bottles, and in a very short time both were completely relieved. The explanation is simply this, that the acid and the hypophosphites in the state of the one stomach was just what was needed, while the nervine and the alkali was just what the other needed. I believe that as far as we possibly can we ought to ascertain what is producing this particular sickness in this particular woman, and not take it for granted that all morning sicknesses are from identically the same cause. In my experience I have seen a great many severe cases. Pregnancy acts differently in different persons, and the thing that would remedy one case would be useless in another. I have seen a good many that I considered very severe cases, but it has been my lot to see one that was of unusual severity, at least the result shows it to have been so.

A lady of whose medical history I know comparatively little came under my observation for four visits. She was several months gone in pregnancy and was suffering with profound morning sickness. A particular characteristic of it, as I noticed in these four visits, which extended over about ten days, was ptyalism. In fact in my presence she was so overcome that she had to vomit. As I was seeing the case in connection with another physician, a friend and relative, who had been there about an hour before my last visit, I did not push the investigation at that time as I had no particular alarm. That was on Thursday evening. It had been the case for the husband to drop in to tell me how she was feeling, and to telephone if she was feeling unusually bad. Thursday evening I saw her last. Sabbath morning the physician dropped in and told me he had called and found her in a very critical condition. It seems that on Friday morning a lady physician had been called in, and on Friday and Saturday had seen her in this condition of increasing irritation of the stomach, and vomiting of some cloudy fluid, and on Sabbath morning when her friend, the physician, saw her, she was in a critical condition, and in spite of dilatation and everything else that could be accomplished on Sabbath night, she died on Monday. I felt impressed at the time and more impressed upon hearing Dr. Blume's paper to-night, that a *post mortem* examination should have been held on the case.

DR. KOENIG: It seems to me that in looking for a specific cause of vomiting in pregnancy we overlook the fact that the nervous system is very powerfully affected by the pregnancy itself. Vomiting in pregnancy is so common that it cannot be due to a gastric lesion; it must depend on an irritation of a nerve ganglion that transmits it to the brain. It has been said that it is impossible to explain why the peculiar motion of a ship should produce vomiting; I have never heard it explained; I do not suppose we can explain it, nor can we explain satisfactorily how the vomiting of pregnancy is produced. It would appear to me that the action of all remedies that are of any value can be accounted for in two ways, one a counter-irritation, which would explain the action of pessaries; explain the action of nitrate of silver when applied to the os; and would explain the action of dilatation. By these an irritation is produced which diverts the attention of nature from the disturbing causes which produce vomiting. The artificial irritation at the os distracts the attention of nature from the other point of irritation, and the vomiting ceases. The other action to which I refer is an anæs thetic action applied to the terminal nerve filaments of the stomach. I have recently seen a remedy, possessing this action, recommended, which Dr. Blume did not refer to in his paper, namely, menthol in two grain doses. Menthol is a powerful anæsthetic, and by deadening the sensibility of the nerves of the stomach I can very readily conceive how the vomiting might be arrested. We all know the tendency of disease is toward recovery, and if vomiting in pregnancy is a disease, it is especially true in this case. The fact accounts for the numerous remedies that are said to be corrective. That fact might account for the results that followed the exchange of remedies in the case alluded to by the President. A large majority of cases recover without medication.

DR. DUFF: I would make the distinction in discussing the subject between simple vomiting during the ordinary morning sickness of pregnancy, the more persistent and aggravated vomiting, and the pernicious vomiting. There is a conservatism of nature in the first class; indeed, some observers declare this to be so. inasmuch as women thus affected go through their pregnancy, as a rule, better than do those who are not. The pernicious vomiting, due entirely to pregnancy, without complication, is, I think, a rarity. The failure up to this time of observers to agree upon any common cause of vomiting during pregnancy, is *prima facie* evidence of varying causes. Our treatment, therefore, should depend upon a rational consideration of each case presented to us. In the cases of Dr. Davis, I do not think that when the exchange of medicine was made the patients were about to get well. I think the exchange was a happy one, inasmuch as the then treatment was in accordance with the acid or alkaline condition of the secretions. I did not notice that the doctor said anything about electric treatment. I think it is sometimes efficacious. Another mode of treatment is the application of cocaine to the cervix uteri. Injecting it into the tissues of the cervix, I think, gives the best results, although painting with a 15 per cent. solution may answer as well.

DR. BLUME: It has been said that pessaries should not be employed to retain a gravid uterus which has been retroflected. Vomiting, as a rule, occurs during the first few months of pregnancy; the replaced uterus will, at so early a period of gestation, often need a support or become again retroflected. We have two means to retain the uterus in its normal position: 1st, Tampons; 2d, pessaries. Tampons must frequently be changed, are therefore inconvenient and even injurious, as they may incite contractions and thus produce abortion. A suitable pessary does no harm, under these circumstances, as I have seen in many instances. Stem-pessaries cannot be considered here. In my opinion they should never be used. They are certainly contraindicated during pregnancy.

Calomel has not been mentioned by me, because it failed entirely to influence the gastric disturbances in my cases. It acts as a simple purgative, and is indicated or may be tried in cases complicated with costiveness.

Gastric medication is applicable only in the milder forms of vomiting. Cases of pernicious vomiting, where everything is rejected by the stomach, cannot be relieved by drugs, be they given by the mouth, by the rectum, or hypodermically. It may sometimes be possible to stop the vomiting for a few hours, but, as a consequence, the nausea becomes so intense that the patient feels relieved as soon as the vomiting commences again.

One gentleman said that dilatation of the cervical canal has been practiced many years ago. This method, first recommended by Copeman, justly bears his name, Copeman's method. Dilatation of the cervix, if carefully effected, does not interrupt pregnancy. But if the internal os is dilated, or, as one gentleman recommended to-night, if the membranes are detached around the internal os, abortion will probably be the consequence.

The view, expressed by one gentleman, that patients with the ordinary vomiting do better at term than those without this disorder, is at least surprising and by no means supported by experience.

In conclusion I wish to touch a point which I have not discussed in my paper, viz.: When has the vomiting become uncontrollable, and when is the induction of abortion indicated? This question is a very important one, for if we wait too long the woman will in all probability lose her life. Uncontrollable vomiting is apparently very rare in our vicinity. The case reported by Dr. Davis is the only one I heard of in this city, and I regret that no autopsy has been made. In New York where this subject has been discussed in the Obstetrical Society, last fall, pernicious vomiting must be a rather frequent complication, as almost every speaker reported cases where artificial abortion had to be induced to save the patient. Several women died, the evacuation of the uterine contents having been too long delayed. All the speakers agreed that abortion should be induced before the condition of the patient had become critical.

I believe that no precise rules can be given as to when to empty the uterus, and that every case must be treated according to its peculiarities. When the various methods of treatment have been tried in vain, when the patient becomes more and more emaciated, it seems hazardous to wait for the most extreme degree of exhaustion. A consultation should be held and the induction of abortion, the last chance of saving the woman's life, should not be postponed too long.

DR. LEMOYNE: In December, 1890, I was requested to see a woman in consultation, who was supposed to be six and a half months pregnant. She has been delivered of two children at full term previously, and had one miscarriage at about six months. For about two weeks she had noticed some swelling of the lower extremities, and a specimen of her urine, which was procured the evening previous to my attendance, was found to be so largely composed of albumen as almost to solidify by boiling. She had taken her evening meal with her family, between six and seven o'clock, but while at the table experienced considerable pain in the abdominal region, and was compelled to retire before finishing. She was assisted to bed, and medical attention procured. Between that time and six o'clock the following morning she had three very decided convulsions. At six o'clock A. M., her expression was rather dull, but she responded intelligently to questions, and recognized persons who addressed her. The mouth of the uterus was sufficiently patulous to admit the point of a finger, which readily detected the body of the fœtus. No instrumental apparatus being at hand for the dilatation of the neck of the uterus, suitable appliances were immediately sent for. But the patient's condition, being such as to promise an early return of the convulsions, admitted of no delay, and dilatation was practiced with great perseverance and determination by means of the fingers. The success of this method was such that when the Bames dilators and parallel steel blades arrived, the divulsion was beyond their capacity, but not sufficient to admit the hand. At 9 A. M., no relief being experienced and every moment seeming to endanger the patient's life, the long obstetrical forceps were resorted to with the intention of either grasping the fœtus in their blades and forcing it away, or effectually dilating the mouth of the uterus by delivering the forceps in the locked position. The first mentioned plan failed, as no engagement could be procured; but with little difficulty the blades were successfully introduced, locked and gradually delivered, dilating the uterus sufficiently to enable the hand to enter, seize the thighs of a breech-presenting fœtus, and accomplish its speedy delivery. Two modified convulsions occurred after the delivery, and five severe ones previous to it. The normal function of the kidneys was soon re-established, and a very satisfactory recovery soon followed.

I offer the history of this case, believing it to illustrate a very valuable and nearly always practicable method of dilating the uterus, and thinking that it may be new to others as it has been to me.

DR. DUFF: The simple introduction of the forceps through the os, locking them without grasping any portion of the child, and withdrawing them for the purpose of dilatation, it appears to me would be impossible except where the head was still above the superior strait, where there was the same condition in a breech, or where there was an oblique presentation. I have frequently introduced the forceps where the os was only dilated sufficiently to admit of their introduction with the double purpose of dilating and of traction. I think Dr. LeMoyne's method justifiable.

DR. BLUME: If delivery of the head should be impossible, I think it is safest to turn by the hand and extract; a woman could be delivered in that way. I do not know in this case whether the child was living or not. I think we have other measures which should be tried first; for instance, anæsthesia.

DR. LEMOYNE: The dilating of the os, I stated in my paper, was done by physical means. I introduced my fingers, first one finger, and then another finger beside it, and finally two fingers and the thumb, until I reached a degree of dilatation that would admit the blades of the forceps consecutively. I also stated that, having no suitable instrument for the purpose when the case was thrust upon my treatment, I had to resort to natural means, and by the time the instruments arrived by which I expected to accomplish dilatation, I had already dilated to a sufficient extent with my fingers to introduce the blades of the forceps, and my diagnosis of the position being still uncertain, I introduced the forceps with the intention of seizing any part which might present. It strikes me as a very fortunate thought, and resulted certainly very favorably.

---

AWAY WITH KOCH'S LYMPH.—I have given Koch's lymph a fair trial and have carefully observed its effects, and have become firmly convinced both of the danger which attends its use and its utter inability to cure any form of tuberculosis. In not a single instance of eleven cases of surgical tuberculosis that came under my own observation did the treatment result in anything more than a temporary improvement, and in several of them it was followed by local extension of the disease and serious impairment of the general health. The effect of tuberculin proved more serious in the treatment of the forty-three cases of pulmonary tuberculosis. There can be but little doubt that in a number of the fatal cases, death was hastened by the treatment, and that in a number of the mild cases it contributed largely towards the rapid local extension of the lesion ; while the tuberculin treatment of pulmonary tuberculosis can show no better results, it is difficult to ignore the fact that it has been productive of more harm than almost any other plan of treatment heretofore suggested, and on this score alone the verdict "Away With Koch's Lymph!" is timely and imperative.—*N. Senn, M. D.*

